# The Knowledge, Attitudes and Usage of Complementary and Alternative Medicine of Medical Students

**DOI:** 10.1093/ecam/nen075

**Published:** 2011-02-14

**Authors:** Dawn DeSylvia, Margaret Stuber, Cha Chi Fung, Shahrzad Bazargan-Hejazi, Edwin Cooper

**Affiliations:** ^1^David Geffen School of Medicine, University of California, Los Angeles, CA 90024-1759, USA; ^2^Charles Drew University of Medicine and Science, Los Angeles, CA, USA

## Abstract

The increasing use of CAM by patients has led to an increase in teaching about CAM in medical school in the US. In preparation for initiation of a new curriculum in Integrative Medicine at the David Geffen School of Medicine at UCLA a cross sectional survey was used to assess medical students': (i) familiarity, (ii) opinions, (iii) personal use and (iv) willingness to recommend specific CAM modalities, using a five point Likert scale of an established measure. A total of 263 first, second and third year medical students at UCLA completed surveys. Third year students reported less personal use of CAM and less favorable attitudes towards CAM than first year students. Since this was a cross-sectional rather than longitudinal study this may be a cohort effect. However, it may reflect the increased curricular emphasis on evidenced-based medicine, and subsequent student dependence on randomized clinical trials to influence and guide practice. This will need to be addressed in curricular efforts to incorporate Integrative Medicine.

Complementary and Alternative Medicine (CAM) has become a $42 billion dollar industry, with 30–50% of Americans utilizing some form of CAM for their healthcare [1]. There is rapidly growing literature about the use of CAM in a variety of medical situations including chronic pain [2], cancer survivors [3], functional bowel disorders [4], stroke [5] and depression [6]. Much of the focus in medical education is on the teaching of Integrative Medicine, which makes use of both conventional and complementary/alternative approaches. The National Center for Complementary and Alternative Medicine of the National Institutes of Health has made recommendations regarding the teaching of Integrative Medicine in medical schools [1, 7, 8]. Specific recommendations have recently been published regarding the content and approaches to such education [9–13]. Medical schools across the United States [14–17] in the United Kingdom [18] and in Israel [19] have done recent needs assessment, indicating an enthusiasm among students for teaching of these topics.

In preparation for such teaching, the medical community around the world is beginning to examine the relationship between medical training and the attitudes of medical students towards Integrative Medicine and CAM. A Canadian study in 2000 found that medical students were less knowledgeable about CAM than students in other medical disciplines [20]. A study of five different medical training programs in 2004-2005 in the United States found that students at allopathic medical schools in the mid-western US were less positive about complementary and alternative modes of treatment than those from allopathic schools in the northwestern US [21]. A paper in 2003 from the United Kingdom suggested that attitudes towards CAM approaches tended to become more negative over the first 3 years of medical school [22]. However, it appears that entering medical students in the United Kingdom may have had a limited exposure to CAM modalities. A more recent longitudinal study in California found that the attitudes of medical students tended to remain relatively consistent over the years of medical training [23].

The David Geffen School of Medicine at the University of California, Los Angeles has been refining the integrative medicine aspect of the medical school curriculum. Based on the studies described above, it appears that measurement of the effectiveness of any such curricular change requires measurement of attitudes as well as knowledge. This study was conducted to establish a baseline of familiarity, attitudes and experience with integrative medicine in current UCLA medical students. This will be used to assess the impact of planned changes in the curriculum in integrative medicine.

## 1. The Survey

A literature search found two existing measures which would fit the purposes of this study. The first measure was the 29 item Integrative Medicine Attitude Questionnaire (IMAQ) which was designed for use with physicians and had a Cronbach's coefficient alpha of 0.83 [24]. The second measure, the 10 item CAM Health Belief Questionnaire (CHBQ), was designed for medical students and was found to have a Cronbach's coefficient alpha of 0.75 [25]. Our survey consisted of the 10 items of the CHBQ, plus most of the IMAQ, along with some demographic questions (year in medical school, age, gender, ethnicity) and two question about self care (diet and exercise). Age was used as a dichotomous rather than continuous variable, to see if “older" students (defined as those over age 29) would differ from those who had come to medical school relatively directly from undergraduate education.

There were three sections to the survey. In the first, respondents answered yes/no about their familiarity, personal use, and willingness to recommend to a friend or patient regarding 15 different modalities of treatment. In the second part, students reported on their use of a variety of information sources for their knowledge about CAM. The third part consisted of 23 questions using a five point Likert Scale assessed their beliefs and attitudes towards CAM.

The survey was made available to all registered first, second and third year medical students in the 2004-2005 academic year at any of the three programs which are a part of the David Geffen School of Medicine at UCLA. The study received Institutional Review Board (IRB) exempt approval from UCLA. In the fall of 2004 an email was sent to students in the classes of 2008, 2007 and 2006 inviting them to participate in an anonymous online survey. A follow-up invitation in January 2005 was extended in person, with paper copies of the survey provided in class after their lectures for first and second year students and after orientation sessions for their clinical rotations for third year students. Power bars were offered as thanks to those who completed the surveys.

Analyses were done using SPSS version 14 software (SPSS, Inc., Chicago, IL, USA). The frequency and distribution of familiarity with, attitudes towards and usage of CAM were examined and compared by year in medical school. The survey had a Cronbach's *α* value of 0.85, similar to that found in the previous use of the component surveys. The total attitude scale mean scores were computed by averaging across all 23 items. Chi square tests were performed to compare between years. Pearson Correlations were used to analyze the association between attitudes towards CAM, year in medical school, personal use, and likelihood to suggest to a friend or patient. All significant results are reported at a *P* < .05. All confidence intervals (CI) are reportable at 95%.

## 2. Results

**Figure 1:**
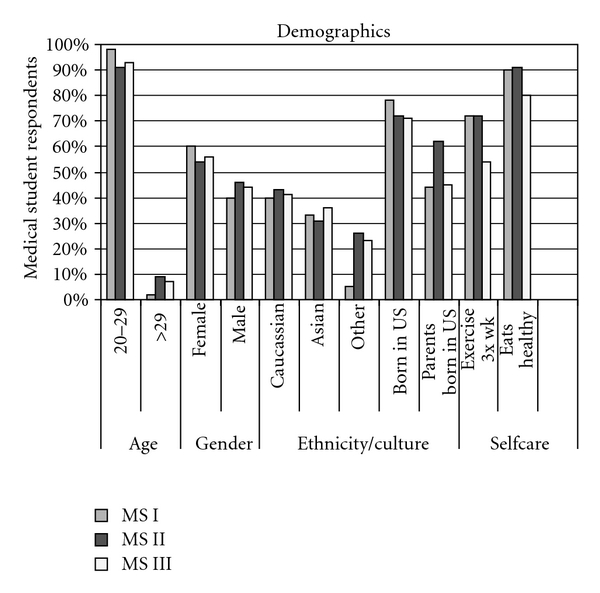
Descriptive statistics on medical student respondents.

The total number of subjects was 261. The response rate was comparable in each of the 3 years (first year *n* = 93/152, 61%; second year *n* = 80/144, 56%; third year *n* = 88/145, 61%), for an overall response rate of 59%.  Figure 1 shows the demographic characteristics of the responding students. Of the demographic variables, only gender and class year were significantly associated with survey results.

**Figure 2:**
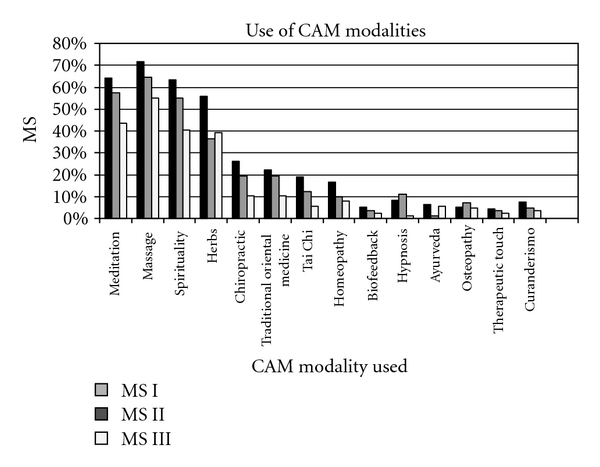
Use of CAM modalities by medical student respondents.

Self-reported use of each of the CAM modalities is shown in Figure 2. Massage (64%), Meditation/Yoga/Relaxation/Imagery (54.6%) and Spirituality/Prayer were most common used. Year in medical school was significantly related to student's report of use of CAM [*F*(2,262) = 9.016, *P* < .01], with reported usage of CAM lower for third year than for first year students.

Familiarity with CAM modalities did not differ significantly by year in medical school. Of the modalities, the least familiar was curanderismo, a Mexican form of folk healing, with 85% of the students reporting they had never heard of this.

Willingness to recommend CAM to friends or patients is reported in Tables 1 and 2. Although there was some relationship between what students have used and what they will suggest, this was not always linearly correlated. For example, almost half of the students reported they would suggest Traditional Oriental Medicine (TOM) to patients, while only about 20% reported use of TOM. On the other hand, more students had used herbs or supplements than would recommend herbs or supplements to a patient. Female students in the first 2 years were more likely to recommend CAM to a friend than their male classmates (*P* = .02 for first years and *P* = .01 for second years). However, there were no differences seen between male and female responses for third year students.

Overall beliefs and opinions about complementary and alternative medicine were not significantly different between the first and second year classes, with a mean score of approximately 3.7 out of five for the 23 questions. However, third year students were more negative on the CAM attitude scale than either first or second year students [*F*(2,258) = 7.283, *P* < .01].

The highest rated attitude items overall are seen in Table 3. They appear to reflect a belief in the physician-patient relationship and the importance of self-care for physicians. The lowest rated items (reverse scored) indicated that medical students have embraced the basic tenets of evidence-based medicine (“Treatments not tested by randomized control trials should be discouraged" and “Physicians should avoid recommending botanical medicines based on observations of long-term use in other cultures and systems of healing, because such evidence is not based on large randomized controlled trials.").

## 3. Discussion

The students at UCLA appear to be familiar with the most commonly used forms of CAM and would be relatively comfortable recommending those with which they are familiar. However, despite the predominance of Hispanic patients in this medical community and the cross-cultural emphasis of this southern California school, very few of the students were familiar with or comfortable with the Mexican folk healers, the curanderismo.

As has been found among medical students in the United Kingdom, female students were more likely to be positive about the use of CAM approaches [26]. This is not consistent with the findings of a study of third year medical students in Florida reported in 2001 [17], and is thus worthy of further investigation.

In this cross-sectional study, the likelihood of using CAM modalities suggesting CAM to others, and positive attitudes towards CAM were all significantly lower in third year medical students than in first year medical students. This may reflect the major curricular change at the David Geffen School of Medicine which began in 2003, and affected the classes of 2007 and 2008 but not the class of 2006. This new curriculum integrated basic and clinical science teaching, changing the way in which integrative medicine was taught, although this was not a specific focus of the change. The new curriculum also added an additional emphasis on clinical reasoning and evidence-based medicine.

The results of the survey have helped to shape the specific goals of the proposed curriculum reform. Although knowledge can clearly be improved, there were some very encouraging findings. Most of the students in all years endorsed an understanding of the importance of patients' spiritual beliefs in healing, and a belief that a strong relationship between patient and physician can be as important as other therapeutic interventions. Students also appeared to believe that a physician who is healthy and balanced is more effective in aiding in the health and balance of another. This belief was mirrored by a majority of the students reporting that they take good care of themselves by exercising and have good eating habits. Unfortunately, medical students often encounter many pressures and models which are not supportive of the importance of self-care, relationships, or outside interests, particularly during the clinical (patient contact) years of training [27] which was seen in the decreased report of self care in third year students. Additional curriculum on CAM alone will not address this issue. However, CAM teaching has been used at some schools to enhance medical students' well-being as well as patient-centered care [28, 29]. This suggests that there are ways to change the context in which medicine is taught as well as the content so that it is compassionate and “student-centered". This will provide nurture for students to retain and carry these qualities into their interactions with patients. It appears that teaching integrative medicine should be integrated with the ongoing efforts in teaching humanistic care and self care and with faculty development. This will be the goal of the proposed new curriculum.

### 3.1. Limitations

There are several significant limitations to this study. We used a convenience sample of medical students at one institution, and thus the results may not be generalizable to other schools. Although the response rate was relatively good for a survey instrument, it was sufficiently small that there is undoubtedly a self-selection bias, which possibly skews the findings. Since the data are cross-sectional rather than longitudinal we do not have information on the actual change in knowledge and attitudes of any specific cohort of students over time.

## Figures and Tables

**Table 1:** Questions endorsed at the highest levels by all 3 years of students.

The spiritual beliefs and practices of patients play no important role in healing, and practices of patients play no important role in healing. (reverse scored)
A strong relationship between patient and physician can equally important as other therapeutic interventions.
Physicians who model a balanced lifestyle (i.e. Attending to their own health, social, family and spiritual needs, as well as interests beyond medicine) generate improved patient satisfaction.
Physicians who strive to understand themselves generate improved patient satisfaction.
Teaching medicine based on a healing model, which integrates the biochemical, psychological, social, and spiritual components of disease, will lead to better patient care.

**Table 2:** Reported likelihood of suggesting CAM to at friend (% of respondents).

CAM modality	First year (*n* = 93)	Second year (*n* = 80)	Third year (*n* = 88)
Meditation/Yoga/ Relaxation/Imagery	68.4	69.5	51.7
Massage	73.7	73.2	57.5
Spirituality	57.9	59.8	43.7
Herbals	55.8	31.7	28.7
Chiropractic	33.7	32.9	16.1
Traditional oriental medicine	43.2	42.7	35.6
T'ai Chi	40	34.1	29.9
Homeopathy	16.8	13.4	12.6
Biofeedback	5.3	17.1	17.2
Hypnosis	16.8	22	5.7
Ayurveda	8.4	7.3	6.9
Osteopathy	16.8	17.1	10.3
Therapeutic touch	11.6	7.3	5.7
Curanderismo	11.6	4.9	2.3

**Table 3:** Likelihood of suggesting CAM to a patient (% of respondents).

Modality	First year (*n* = 93)	Second year (*n* = 80)	Third year (*n* = 88)
Meditation/Yoga/ Relaxation/Imagery	67.4	76.8	61.4
Massage	69.5	72	56.8
Spirituality	48.4	58.5	38.6
Herbals	38.9	26.8	29.5
Chiropractic	34.7	39	14.8
Traditional oriental medicine	41.1	50	30.7
T'ai Chi	43.2	35.4	34.1
Homeopathy	13.7	11	13.6
Biofeedback	11.6	15.9	33
Hypnosis	17.9	19.5	15.9
Ayurveda	10.5	7.3	5.7
Osteopathy	18.9	19.5	17
Therapeutic touch	10.5	11	9.1
Curanderismo	4.2	3.7	2.3
